# The complete plastid genome and phylogenetic analysis of *Gracilaria spinulosa*

**DOI:** 10.1080/23802359.2019.1642162

**Published:** 2019-07-17

**Authors:** Tao Liu, Xianming Tang, Xuli Jia, Xiangyu Wu, Min Huang, Jun Zeng, Weizhou Chen

**Affiliations:** a Hainan Academy of Ocean and Fisheries Sciences, Haikou, People’s Republic of China;; b Hainan Provincial Key Laboratory of technology for tropical seawater aquaculture, Haikou, People’s Republic of China;; c Laboratory of Genetics and Breeding of Marine Organism, College of Marine Life Sciences, Ocean University of China, Qingdao, People’s Republic of China;; d Marine Biology Institute, Shantou University, Shantou, People’s Republic of China

**Keywords:** *Gracilaria spinulosa*, plastid genome, phylogenetic analysis, Gracilariaceae

## Abstract

*Gracilaria spinulosa* is an economical species of marine red algae. The length of its plastid genome sequence is 179,082 bp; a total of 236 genes were determined, including 203 protein-encoding genes, 3 rRNA genes, 29 tRNA genes, 1 ribonuclease gene, and 1 intron inserted into the *trnM* gene. The gene content and structure of Gracilariaceae species were relatively well conserved. Phylogenetic analysis showed that *G. spinulosa* had a closer relationship with *Gracilaria salicornia* in *Gracilaria.* The complete plastid genome sequence provided will help the understanding of *Gracilaria* evolution.

*Gracilaria spinulosa* (Okamura) Chang & B.M.Xia is a marine red alga belonging to the family Gracilariaceae. *Rhodymenia spinulosa* (Okamura) is the basionym of *Gracilaria spinulosa*. It is an agar-producing seaweed (Lin 2006). Previous studies focused on the vegetative and reproductive morphology and taxonomic status analyses of *G. spinulosa* (Withell et al. 1994; Lin 2006). It was originally described from Taiwan, with bushy and erect Thalli, irregularly dichotomous branches, flattened blades, a discoid holdfast, and occasionally with a short stipe (1–2.5 mm long; Lin 2006). However, no previous genomic studies on *G. spinulosa* have been reported.

Herein, we report the determination of the *G. spinulosa* plastid genome sequence. The specimen was collected from Yinggehai, Hainan Province (18°30′36′′ N, 108°42′15′′ E) was sequenced, and was deposited at the Culture Collection of Seaweed at the Ocean University of China (accession number: 2017060066). Total DNA was extracted via the modified CTAB method (Doyle and Doyle 1990). Paired-end reads were sequenced by using Illumina HiSeq system (Illumina, San Diego, CA, USA). Approximately 27 Gb of paired-end (150 bp) sequence data were randomly extracted from the total sequencing output and used as input for NOVOPlasty (Dierckxsens et al. 2017) to assemble the plastid genome. The complete plastid genome, using *Gracilaria tenuistipitata* var. *liui* (AY673996) as the seed sequence, was annotated with Geneious R10.1.3. The tRNA genes were identified using tRNAscan-SE Search Server (Schattner et al. 2005).

The complete *G. spinulosa* plastid genome is a circular DNA molecule measuring 179,082 bp in length, and the overall A + T content of the complete plastid genome was 71.3% (GenBank accession number MN053319). The plastid genome contained 236 genes, including 203 protein-coding, 3 rRNA, and 29 tRNA genes, 1 ribonuclease gene (*rnpB*), and 1 intron interrupting the *trnM* gene. The length of the coding region was 145,035 bp, corresponding to 81.0% of the total length. The plastid genome of *G. spinulosa* was compact, with 10 pairs of overlapping genes found with overlap lengths of 3–95 bp (*rpl23*–*rpl4*, *rpl14*–*rps17*, *trnT*–*ilvB*, *ycf40*–*rps1*, *ycf29*–*trnH*, *psbD*–*psbC*, *carA*–*ycf53*, *chlI*–*trnR*, *atpF*–*atpD*, and *rps18*–*rpl33*). The gene numbers and structures were largely similar among Gracilariaceae species published in the NCBI sequence database; their plastid genomes were relatively well conserved, with no gene rearrangement phenomena.

Phylogenetic analysis was conducted using 82 shared plastid protein sequences from 17 red algal plastid genomes, using *Cyanidioschyzon merolae* as an outgroup. The nucleotide sequences were aligned by using MAFFT (Katoh et al. 2002). Concatenated alignments were generated and poorly aligned regions were removed by using the Gblocks server (http://phylogeny.lirmm.fr/phylo_cgi/one_task.cgi?task_type=gblocks) (Castresana 2000). MrBayes 3.1.2 software was used to construct the amino acids phylogenetic tree (Ronquist and Huelsenbeck 2003). The results showed that all red algal taxa were clearly separated according to their original class (Figure 1). The *Gracilaria* species formed a branch, in which *G. spinulosa* showed a closer relationship with *Gracilaria salicornia.* The determination of the complete plastid genome sequence will help the understanding of *Gracilaria* evolution.

**Figure 1.**
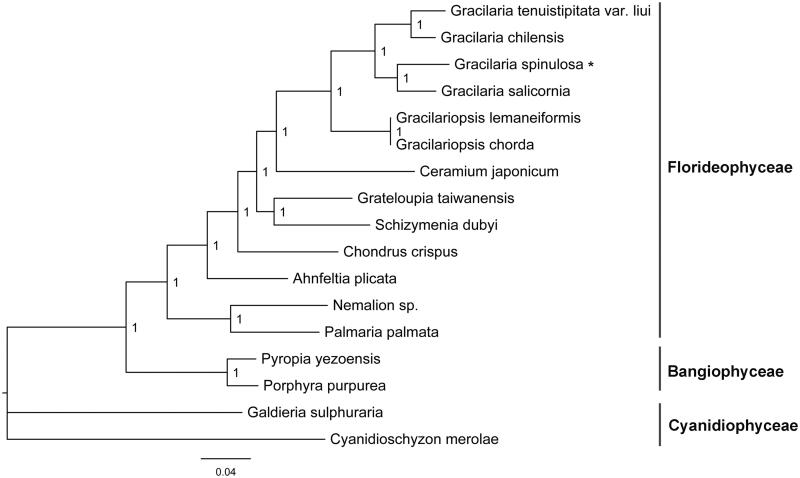
Phylogenetic tree (Bayesian method) based on the complete plastid genome sequence of red algae as shown below: *Gracilaria spinulosa* (MN053319), *Gracilaria salicornia* (NC_023785), *Gracilaria tenuistipitata* var. *liui* (AY673996), *Gracilaria chilensis* (NC_029860), *Gracilariopsis chorda* (NC_031149), *Gracilariopsis lemaneiformis* (KP330491), *Grateloupia taiwanensis* (KC894740), *Schizymenia dubyi* (NC_031169), *Chondrus crispus* (NC_020795), *Ceramium japonicum* (NC_031174), *Nemalion* sp. (LT622871), *Ahnfeltia plicata* (NC_031145), *Palmaria palmata* (NC_031147), *Pyropia yezoensis* (KC517072), *Porphyra purpurea* (U38804), *Galdieria sulphuraria* (KJ700459), and *Cyanidioschyzon merolae* (NC_004799). The asterisks after species names indicate newly determined plastid genomes.
